# Decoding poxvirus genome

**DOI:** 10.18632/oncotarget.5892

**Published:** 2015-09-28

**Authors:** Zhilong Yang, Bernard Moss

**Affiliations:** Division of Biology, Kansas State University, Manhattan, Kansas, USA

**Keywords:** poxvirus, vaccinia virus, translatome

Deciphering the information encoded in genomic sequences is a key step in modern biomedical research. Recent findings indicate that this endeavor can be far more complex than anticipated, even for relatively small viral genomes. Vaccinia virus, the prototypic member of the poxvirus family, was initially annotated to have approximately 200 open reading frames (ORFs) of 65 or more amino acids within its 200 kbp double-stranded DNA genome. This annotation has framed the molecular biological studies of vaccinia virus since then. To further decode information in the vaccinia virus genome, we carried out systematic genome-wide ribosome profiling recently published in the Journal of Virology [1]. In ribosome profiling, only the mRNA fragments bound and protected by ribosomes are analyzed by next generation sequencing, which can quantify active protein translation with superb sensitivity, resolution and clarity [2]. We confirmed that the majority mRNAs of previously annotated ORFs are actively translated, although at greatly different frequencies. In addition, even though the long transcripts made during the late stages of infection read through adjacent ORFs, only the first is translated.

The most intriguing finding of this study was the existence of numerous unsuspected translation initiation sites, which were revealed by ribosome profiling of infected cells treated with the translation inhibitor Harringtonine or Lactimydomycin. Both inhibitors arrest the ribosomes at or near the translation initiation site on mRNA with single nucleotide resolution. In addition to the start sites of most annotated ORFs, approximately 600 additional putative translational initiation sites using AUG or alternative near-cognate codons were discovered. Most of these ORFs are embedded in previously annotated ones including truncated non-frameshifting ORFs and downstream frameshifting ORFs. Others are located in non-coding regions including a few upstream ORFs located in the 5′ untranslated region of mRNA, ORFs in intergenic regions and antisense-strand of the annotated ORFs. These findings suggest multifaceted translation of the vaccinia virus genome. In concert with our earlier findings of pervasive transcription [3,4], these studies demonstrate that vaccinia virus encodes many more products than previously recognized, although their biological relevance is yet to be determined.

Most of the newly identified ORFs are small in size, as they were below the previous arbitrary cut-off of 65-amino-acids. In fact, several recent studies have suggested that small ORFs encoded by vaccinia virus can perform essential functions in viral replication. The smallest protein characterized has only 35 amino acids and is required for virus entry into cells [5]. Pervasive transcription and translation apparently increase the coding capacity and expand the functional repertoire of poxvirus genomes; some of the newly identified ORFs may have important structural and regulatory functions. From an evolutionary perspective, pervasive transcription and translation may enable poxviruses to express new genes from endogenous or horizontally acquired cellular DNA that is randomly inserted into the viral genome. This plasticity may allow poxviruses to adapt to environmental changes. In fact, poxviruses acquired many cellular genes during their evolution, presumably by reverse transcription of mRNA since introns are absent. Though many questions remain regarding the functions of these novel identified ORFs in vaccinia virus, other viruses such as cytomegalovirus [6] as well as eukaryotic cells also encode many more gene products than previously recognized [7].

It is possible that these newly identified translation products contribute to the differences in host range and pathogenicity of poxviruses. The virus responsible for smallpox is human-specific and was responsible for more deaths than all other infectious diseases combined in history. Although eradicated three decades ago, the smallpox virus can still be potentially used as biological weapons from unsecured stocks, genetic engineering or *de novo* synthesis. Other poxviruses, such as monkeypox and molluscum contagiosum virus, also cause serious problems in humans. Host range and pathogenicity also contribute to the specificity and efficacy of poxviruses as oncolytic agents in combating cancers and as vaccine vectors for prevention of infectious diseases. There are numerous licensed veterinary vaccines based on recombinant poxviruses that express genes of other pathogens and clinical trials to prevent infection with HIV, influenza virus, and malaria are ongoing. Poxviruses are powerful tools in studying immune system as they encode many genes that engage numerous aspects of innate and adaptive immune systems. A better understanding of the roles of the newly identified ORFs may provide insights into poxvirus replication and help to develop strategies to prevent poxvirus infection and improve poxviruses as oncolytic agents and vaccine vectors.
